# Nc‐RNA‐mediated low expression of AZIN1 correlated with unfavorable prognosis in kidney renal clear cell carcinoma

**DOI:** 10.1002/cam4.70105

**Published:** 2024-08-14

**Authors:** Xinyi Zeng, Shen Du, Lou Geng, Jiexian Ma

**Affiliations:** ^1^ Department of Hematology Huadong Hospital Affiliated with Fudan University Shanghai China; ^2^ Department of Nuclear Medicine, Zhongshan Hospital Fudan University Shanghai China; ^3^ Department of Hematology, Institute of Hematology, Shanghai Changhai Hospital Naval Medical University Shanghai China

**Keywords:** AZIN1, immune infiltration, KIRC, ncRNA

## Abstract

**Objective:**

Kidney renal clear cell carcinoma (KIRC, ccRCC) is the most common type of renal cancer with high recurrence and mortality. It has long been recognized that Antizyme inhibitor 1 (AZIN1) serves as a pro‐oncogenic molecule in multiple cancers. However, the clinicopathological features of AZIN1 in KIRC remain unexplored.

**Materials and Methods:**

The Cancer Genome Atlas (TCGA, TIMER, and GEPIA) were employed for pan‐cancer expression and survival analysis of AZIN1, indicating the unique anti‐tumor role of AZIN1 in KIRC. The expression and clinical characteristics of AZIN1 in KIRC were further proven via Human Protein Atlas and TCGA. single‐sample GSEA was employed to investigate the immune infiltration of AZIN1. Then the downstream pathways were illustrated via the LinkedOmics, Metascape, and Cytoscape databases. The possible upper regulating noncoding RNAs (ncRNAs) were analyzed from five programs‐TargetScan, StarBase, miRanda, PITA, and miRmap.

**Results:**

AZIN1 is downregulated in KIRC patients. Lower levels of AZIN1 were linked with unfavorable outcomes in KIRC patients. The AZIN1 expression was positively related to immune cell infiltration in KIRC. We also elucidated a possible upstream regulatory ncRNA of AZIN1 in KIRC namely STK4‐AS1/AC068338.2‐miR‐106b‐5p‐AZIN1 axis as well as the downstream signaling pathways.

**Conclusion:**

This study illustrated the unique anti‐tumor role of AZIN1 in KIRC and provided potential value for guiding immunotherapy and targeted therapy.

## Graphical abstract

Graphic abstract of STK4‐AS1/AC068338.2‐miR‐106b‐5p‐AZIN1 axis in carcinogenesis of KIRC.

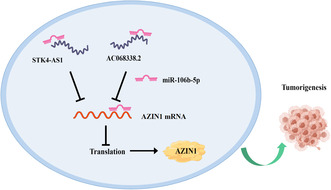

## 1 INTRODUCTION

Antizyme inhibitor 1 (AZIN1) serves as a crucial mediator in polyamine metabolism. Antizyme inhibitor (AZI) increases the ornithine decarboxylase (ODC) activity, accelerating intracellular polyamine biosynthesis.^1^ Polyamines, which include spermidine, putrescine, and spermine, are crucial amino acid metabolites participating fundamental intracellular processes.^2^ Polyamine metabolism is frequently dysregulated in many neoplastic states. Elevated polyamines can play a major role in carcinogenesis via participating in cancer‐driving signaling pathways‐ PI3K‐AKT, RAS, and WNT pathways etc.^3^ However, recent studies indicate that polyamines have also been reported in regulating the antitumor response.^4^, ^5^ Given that AZIN1 participates in polyamine metabolism, AZIN1 has been mostly recognized as a pro‐oncogenic molecule in cancers.^6^

Kidney renal clear cell carcinoma (KIRC), which is featured as metabolic reprogramming, accounts for approximately 70%–80% of all subtypes of renal cancers.^7^ As KIRC is insensitive to tranditional radiotherapy and chemotherapy, surgical treatment, targeted therapy, and immunotherapy have brought clinical benefits for KIRC patients. Yet KIRC patients are still at high risk of stepping into relapse or metastasis stages after the initial nephrectomy, which is associated with high mortality.^8^, ^9^ Limited options left KIRC patients with poor prognosis. Therefore, finding new biomarkers not only can provide a better understanding of the molecular mechanisms but also is the key to implementing individualized treatment and predicting the prognosis of KIRC.

In the current study, we first showed that AZIN1 is downregulated in KIRC patients and high AZIN1 is associated with favorable clinical outcomes in KIRC. Next, the upperstream noncoding RNAs (ncRNA)‐associated regulation of AZIN1 was explored in KIRC. Moreover, the relationships of AZIN1 expression with biomarkers of immune cells as well as immune cell infiltration in KIRC were illustrated. In conclusion, this novel anti‐tumor role of AZIN1 in KIRC was identified in this study.

## 2 MATERIALS AND METHODS

### 2.1 TIMER data mining and GEPIA database analysis

The mRNA expression data of 32 cancer types were obtained from TIMER1.0 (https://cistrome.shinyapps.io/timer/) based on The Cancer Genome Atlas (TCGA). The differential expression analysis of AZIN1 was then performed on the normalized data. The correlation of AZIN1 expression with immune cell infiltration level in KIRC was also analyzed by TIMER.^10^

GEPIA (http://gepia.cancer‐pku.cn/) was employed for gene expression between cancer and normal tissues,^11^ as well as for the prognostic analysis of candidate AZIN1 in KIRC. Univariate analysis of AZIN1 in clinical KIRC samples was performed via GEPIA.

### 2.2 Immunohistochemistry analysis

The immunohistochemical analysis of AZIN1 levels in KIRC and normal tissues was directly obtained from Human Protein Atlas (HPA, https://www.proteinatlas.org/).

### 2.3 Patient samples

We used the KIRC samples from Huadong Hospital Affiliated to Fudan University in June 2024. The study protocols were approved by the ethical committee of Huadong Hospital (approval number: 2024K178). The patients provided written informed consent. This clinical investigation was conducted according to the principles of the Declaration of Helsinki.

### 2.4 Cell culture

Non‐cancer kidney cell line (HA1E), KMRC1, and OSRC2 were cultured in DMEM (Dulbecco's Modified Eagle Medium) and UMRC3 were mainteined in minimal essential medium. All cell lines were supplemented with 10% fetal bovine serum, 2 mmol/L L‐glutamine and 100 IU/mL penicillin/streptomycin. All cells were cultured at 37°C in a humidified atmosphere with 5% CO_2_.

### 2.5 RNA extraction and real‐time polymerase chain reaction

Total RNA was isolated from cells using an RNA isolation kit (Beyotime, Shanghai, China) following the manufacturer's instructions. Then the extracted RNA was reverse‐transcribed to complementary DNA using the PrimeScript RT reagent kit (Takara Bio, Kusatsu, Japan). Quantitative real‐time PCR was applied using a TB Green Premix Ex Taq II kit (Takara Bio). Glyceraldehyde‐3‐phosphate dehydrogenase (GAPDH) was used as an internal control. Each experiment was performed in triplicate. Differences in gene expression level, expressed as fold changes, were calculated using the 2−ΔΔCt method. The forward and reverse primers for GAPDH were 5′‐TGACTTCAACAGCGACACCCA and 5′‐CACCCTGTTGCTGTAGCCAAA, respectively. The forward and reverse primers for AZIN1 were 5′‐CGGAAGTGATGAACCAGCCTTC and 5′‐GGCTGCTTGTAAACAGAGGCTC, respectively.

### 2.6 Immune infiltration analysis by single‐sample GSEA

The GSVA R package was employed for single‐sample GSEA (ssGSEA). Relative tumor infiltration levels of 24 immune cell types were quantified by interrogating expression levels of genes in published signature gene lists.^12^ Infiltration levels were compared between AZIN1 high‐ and low‐groups.

### 2.7 Analysis of gene cluster identification and associated pathways of AZIN1

The LinkedOmics database was introduced to obtain the different‐expressed genes (DEG) of AZIN1. Then Using the Metascape database and Cytoscape, and protein cluster analysis of DEGs was conducted using a molecular complex detection plug‐in to explore key signaling pathways in biological networks and the protein–protein interaction network.

### 2.8 Candidate miRNA and lncRNA screening

Upstream MicroRNAs (miRNAs) of AZIN1 were predicted by five target gene prediction bioinformatic programs‐TargetScan, StarBase, miRanda, PITA, and miRmap. The candidate miRNAs of AZIN1 which appeared in all five platforms were able to be included in further analyses. Besides, StarBase (http://starbase.sysu.edu.cn/)^13^ was applied for expression correlation of miRNA‐AZIN1, as well as prediction of upperstream lncRNAs of miR‐106b‐5p and its expressions in KIRC and normal control.

### 2.9 Statistical analysis

We employed R software (version 4.0.0) with appropriate packages and GraphPad Prism v8.0 software. The comparison of two groups was performed by a two‐tailed student's *t*‐test. The Kaplan–Meier method and log‐rank test were used for survival analysis in patients. The optimum cut‐off value for eight lncRNAs expression was generated using the survfit function via the R package “survminer.” All other cut‐off values were showed as medians. *p*‐value <0.05 was considered as statistically significant.

## 3 RESULTS

### 3.1 AZIN1 is downregulated in KIRC patients and high AZIN1 is associated with favorable clinical outcomes in KIRC

**FIGURE 1:**
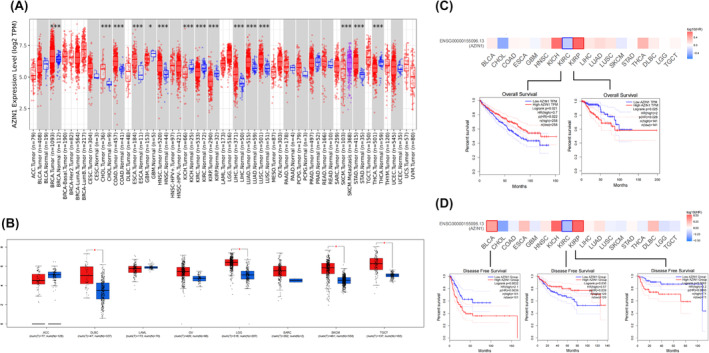
The expression and survival analysis of Antizyme inhibitor 1 (AZIN1) between multiple cancers. (A) AZIN1 expression in tumor and normal tissues in 34 types of cancers via the TIMER database. (B) AZIN1 expression between tumor and normal tissues in eight types of cancers via GEPIA database. (C) The overall survival (OS) plot of statistical significance of AZIN1 in Kidney renal clear cell carcinoma and KIRP. (D)The DFS plot of statistical significance of AZIN1 in BLCA, KIRC, and KIRP. *p < 0.05, **p < 0.01, and ***p < 0.001.

The pan‐cancer analysis of AZIN1's expression (TPM standardized data) was shown in Figure 1A using TIMER 1.0. AZIN1 mRNA levels were markedly downregulated in five types of cancer, including GBM, THCA, KICH, KIRC, and KIRP. However, the mRNA expressions of AZIN1 were significantly higher in BRCA, CHOL, COAD, ESCA, HNSC, LIHC, LUAD, LUSC, and STAD when compared with normal tissues. High expression of AZIN1 was also observed in SKCM metastatic patients. For those cancers lacking normal tissues in the TIMER dataset, we employed the GEPIA database to illustrate the AZIN1 gene expression. These data showed a significant decrease in AZIN1 expression in DLBC, LGG, SKCM, and TCGT. (Figure 1B).

These data showed that the AZIN1 expression levels seem to vary depending on the cancer type, indicating its diverse roles in cancer. Interestingly, AZIN1 was lower in all types of kidney cancer, as seen in KICH, KIRC, and KIRP.

Next, the prognostic value of AZIN1 was validated in those cancers using GEPIA. Patients from each cancer type were then divided into high‐ and low‐expression groups based on the median AZIN1 expression. The results revealed that low AZIN1 expression was associated with an unfavorable overall survival (OS) in KIRC. However, AZIN1's downregulation was associated with better OS in KIRP (Figure 1C). No statistical significance of AZIN1 expression was observed in other cancer types.

For disease‐free survival (DFS), lower expression of AZIN1 in BLCA and KIRP indicated longer DFS, but KIRC patients with lower AZIN1 expression indicated poorer clinical outcomes (Figure 1D), which is consistent with AZIN1's prognostic role of OS in KIRC. Given the above, decreased expression of AZIN1 was identified in KIRC patients, which was correlated with unfavorable prognosis by a combination of OS and DFS. Taken together, AZIN1 seems to play an anti‐tumor role and serves as a favorable prognostic factor in KIRC.

### 3.2 AZIN1 is downregulated in KIRC samples

**FIGURE 2:**
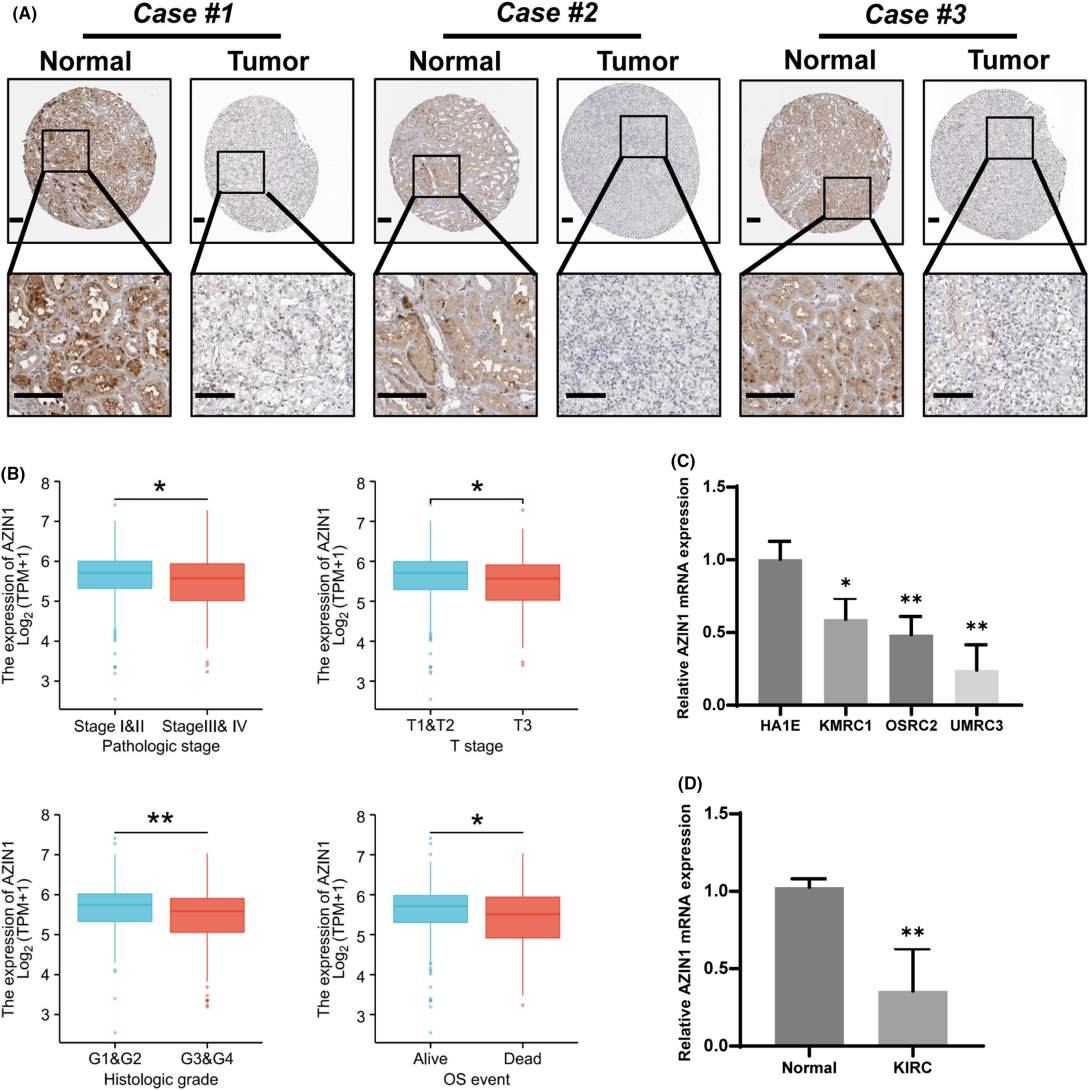
Antizyme inhibitor 1 (AZIN1) expression in clinical samples in kidney renal clear cell carcinoma (KIRC). (A) Immunohistochemistry analysis in tumor tissues and corresponding normal tissues is shown by HPA. Scale bars: 100 μM (B) AZIN1 expression between different stages and associated with clinical outcome in KIRC patients using TCGA database. (C) Levels of AZIN1 mRNA expression in a non‐cancer kidney cell line (HA1E) and three KIRC cell lines (KMRC1, OSRC2, and UMRC3). (D) Real‐time PCR analysis of AZIN1 gene expression in the tumor samples and paired normal tissues of seven KIRC patients. *p < 0.05, and **p < 0.01.

We further confirmed the protein expression of AZIN1 in KIRC patients and normal tissues are illustrated using HPA. The immunohistochemical analysis showed that compared with three normal tissues, AZIN1 protein expressions were low in three KIRC tissues (Figure 2A). Low levels of AZIN1 protein expression were negatively correlated with advanced clinicopathological features in KIRC (Figure 2B). Based on the TCGA database, patients with stage III/IV RCC exhibited lower levels of AZIN1 expression than patients with I/II disease. T stage and histologic grade showed consistent AZIN1 expression in KIRC. In terms of OS, KIRC survivors showed higher levels of AZIN1 than patients who died of KIRC. We then tested the AZIN1 mRNA expression via qPCR in HA1E and three KIRC cell lines (KMRC1, OSRC2, and UMRC3) in Figure 2C. The mRNA levels of AZIN1 in clinical specimens from the tumor samples and paired normal tissues of seven patients with KIRC are shown in Figure 2D.

Furthermore, univariate Cox regression analysis showed that AZIN1, TNM stage, serum calcium, and hemoglobin levels were significantly associated with OS in KIRC patients (*p* < 0.05; Table 1). Besides, T and M stage, as well as AZIN1 levels were independent prognostic factors for OS in KIRC patients sourced from the TCGA database in the multivariate Cox regression analysis (*p* < 0.05; Table 1).

**TABLE 1:** Univariate and multivariate Cox regression analyses of AZIN1 expression and other clinical pathological factors for OS.

Characteristics	Total (N)	Univariate analysis		Multivariate analysis	
		Hazard ratio (95% CI)	*p*‐value	Hazard ratio (95% CI)	*p*‐value
Pathologic T stage	541		**<0.001**		
T1 and T2	350	Reference		Reference	
T3 and T4	191	3.210 (2.373–4.342)	**< 0.001**	2.349 (1.425–3.873)	**<0.001**
Pathologic N stage	258		**0.001**		
N0	242	Reference		Reference	
N1	16	3.422 (1.817–6.446)	**< 0.001**	1.005 (0.307–3.294)	0.993
Pathologic M stage	508		**< 0.001**		
M0	429	Reference		Reference	
M1	79	4.401 (3.226–6.002)	**< 0.001**	4.549 (2.597–7.969)	**<0.001**
Serum calcium	367		**0.001**		
Low	204	Reference		Reference	
Normal	153	1.225 (0.865–1.735)	0.254	0.687 (0.403–1.171)	0.167
Elevated	10	4.846 (2.404–9.769)	**<0.001**	0.571 (0.146–2.230)	0.420
Hemoglobin	461		**<0.001**		
Low	264	Reference		Reference	
Normal	192	0.430 (0.302–0.613)	**<0.001**	0.653 (0.374–1.140)	0.134
Elevated	5	2.663 (0.844–8.400)	0.095	2.263 (0.292–17.542)	0.434
AZIN1	541		**0.007**		
Low	270	Reference		Reference	
High	271	0.663 (0.490–0.896)	**0.007**	0.590 (0.368–0.947)	**0.029**

The bold values indicates *P* values with statistical significance.

Taken together, we further proved that low expression of AZIN1 in KIRC was linked with unfavorable clinical outcomes, indicating the antitumor character of AZIN1 in KIRC. We then investigated the further regulatory mechanism of AZIN1 in KIRC.

### 3.3 AZIN1 downstream signaling pathway in KIRC

**FIGURE 3:**
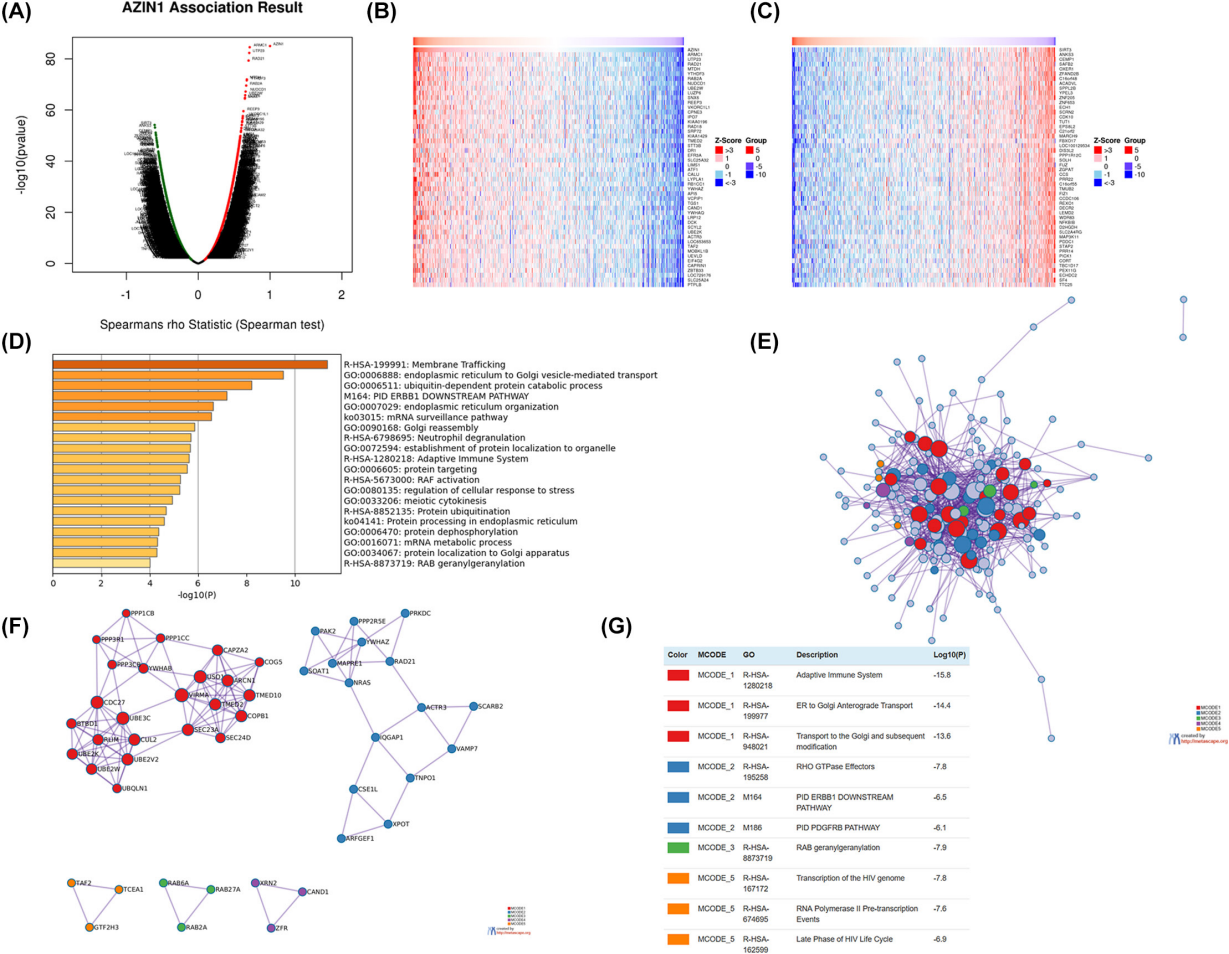
Antizyme inhibitor 1 (AZIN1) downstream signaling pathways. (A) Associated genes of AZIN1 are displayed via linkedomics. (B) Positively correlated genes of AZIN1 are shown in the heatmap. (C) Negatively correlated genes of AZIN1 are shown in the heatmap. (D) Metascape database was used to analyze the GO enrichment results of differentially expressed genes screened based on AZIN1 expression. (E) Cytoscape was used to show the correlated genes of AZIN1. (F) Functional networks of AZIN1 were illustrated in modes. (G) Functional networks of AZIN1 were described.

To investigate the possible downstream signaling pathways of AZIN1, the linkedomics database was used to identify associated genes of AZIN1 in KIRC (Figure 3A). The heatmap for positively correlated genes was showed in Figure 3B while negatively correlated genes were illustrated in Figure 3C. Then the enrichment analysis of positively enriched genes was performed using Metascape. Protein post‐translational modifications (ER to Golgi transport, ubiquitin‐dependent protein catabolic process) were highly enriched (Figure 3D and Table 2), which is consistent with the primary role of AZIN1 in metabolism. Moreover, the results also highlighted that AZIN1 influences the adaptive immune system and neutrophil degranulation. The top 200 positively correlated genes were then depicted with shared functional networks in the same color using Cytoscape as illustrated in Figure 3E–G, including the adaptive immune system and ER‐to‐Golgi transport system, etc. Interestingly, AZIN1 seems to negatively participate in certain metabolic and immune pathways, such as peptidyl‐lysine modification and antigen‐processing events (Figure S1 and Table S1), indicating AZIN1 might regulate the metabolic and immune signaling in a more sophisticated manner.

**TABLE 2:** Functional enrichment analysis of positively correlated genes.

GO	Category	Description	Count	%	Log10 (P)	Log10 (q)
R‐HSA‐199991	Reactome Gene Sets	Membrane trafficking	25	12.50	−11.34	−6.97
GO:0006888	GO Biological Processes	Endoplasmic reticulum to golgi vesicle‐mediated transport	12	6.00	−9.52	−5.76
GO:0006511	GO Biological Processes	Ubiquitin‐dependent protein catabolic process	21	10.50	−8.21	−4.62
M164	Canonical Pathways	PID ERBB1 downstream pathway	9	4.50	−7.19	−3.79
GO:0007029	GO Biological processes	Endoplasmic reticulum organization	8	4.00	−6.61	−3.35
ko03015	KEGG Pathway	mRNA surveillance pathway	8	4.00	−6.54	−3.35
GO:0090168	GO Biological Processes	Golgi reassembly	3	1.50	−5.85	−2.75
R‐HSA‐6798695	Reactome Gene Sets	Neutrophil degranulation	15	7.50	−5.71	−2.67
GO:0072594	GO Biological Processes	Establishment of protein localization to organelle	14	7.00	−5.67	−2.67
R‐HSA‐1280218	Reactome Gene Sets	Adaptive immune system	19	9.50	−5.61	−2.63
GO:0006605	GO Biological Processes	Protein targeting	12	6.00	−5.55	−2.59
R‐HSA‐5673000	Reactome Gene Sets	RAF activation	5	2.50	−5.27	−2.35
GO:0080135	GO Biological Processes	Regulation of cellular response to stress	17	8.50	−5.24	−2.34
GO:0033206	GO Biological Processes	Meiotic cytokinesis	3	1.50	−4.92	−2.11
R‐HSA‐8852135	Reactome Gene Sets	Protein ubiquitination	6	3.00	−4.67	−1.91
ko04141	KEGG Pathway	Protein processing in endoplasmic reticulum	8	4.00	−4.58	−1.85
GO:0006470	GO Biological Processes	Protein dephosphorylation	10	5.00	−4.37	−1.7
GO:0016071	GO Biological Processes	mRNA metabolic process	16	8.00	−4.33	−1.67
GO:0034067	GO Biological Processes	Protein localization to golgi apparatus	4	2.00	−4.29	−1.66
R‐HSA‐8873719	Reactome Gene Sets	RAB geranylgeranylation	5	2.50	−4	−1.44

### 3.4 Correlation analysis between mRNA expression of AZIN1 and immune infiltration

**FIGURE 4:**
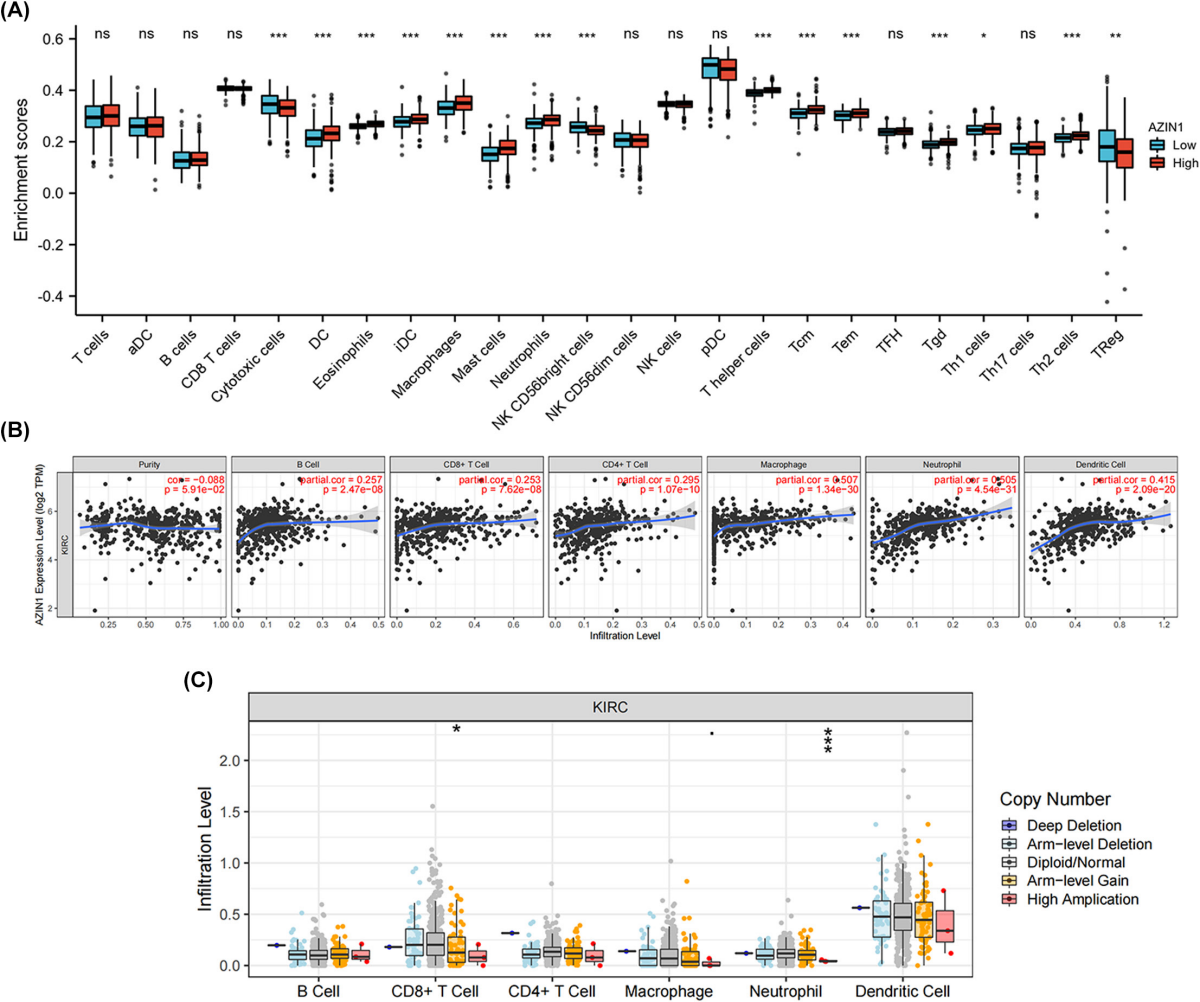
The relationship of immune cell infiltration with Antizyme inhibitor 1 (AZIN1) level in kidney renal clear cell carcinoma (KIRC) (A) Enrichment analysis of immune cells between high and low AZIN1 expression. (B) The correlation of AZIN1 expression with B cell, CD8^+^ T cell, CD4^+^ T cell, macrophage, neutrophil, or dendritic cell infiltration level in KIRC. (C) The infiltration level of various immune cells under different copy numbers of AZIN1 in KIRC. ns, not significant. *p < 0.05, **p < 0.01, and ***p < 0.001.

Polyamines have been knowed to be associated with immune system regulation and altered polyamine metabolism may lead to immune suppression or anti‐tumor immune response depending on the cancer type. The prognosis of KIRC is closely related to immune infiltration and activation of immune cells.^14^ AZIN1 is essential for polyamine homeostasis. AZIs have been considered to function as positive regulators of polyamine levels. The correlation between AZIN1 expression and markers of immune cells was then further validated. With the ssGSEA algorithm, we analyzed the tumor immune microenvironment of high and low AZIN1 groups in the TCGA cohort. (Figure 4A). Most immune cells were highly infiltrated in the low‐AZIN1 group, such as Treg cells, NK CD56 bright cells, and cytotoxic cells, whereas macrophages, mast cells, DC cells, neutrophils, Tcm, Tem, Tgd, T helper cells, Th1 cells, and Th2 cells were highly enriched in the high‐AZIN1 group. The correlation between AZIN1 levels and immune cell infiltration levels was also analyzed. As illustrated in Figure 4B, AZIN1 expression was positively associated with all analyzed immune cells, including macrophage, neutrophil, dendritic cells, as well as B cells, CD8^+^ T cells, and CD4^+^ T cells in KIRC. The significant change in infiltration level of CD8 T cells and neutrophils under various copy numbers of AZIN1 in KIRC was observed (Figure 4C). As provided in Table 3, AZIN1 was significantly positively correlated with M2 macrophage's biomarkers (CD163, VSIG4, and MS4A4A), CD4^+^ T cell's biomarkers, monocyte's biomarkers (CD86 and CSF1R), M1 macrophage's biomarkers (NOS2 and PTGS2), neutrophil's biomarkers (ITGAM and CCR7), and dendritic cells' biomarkers (HLA‐DRA, HLA‐DPA1, HLA‐DPB1, NRP1, and CD1C). It showed that KIRC samples displayed a significant increase of monocytes, macrophages, DC, and neutrophils than the control. These results suggested AZIN1 might inhibit carcinogenesis of KIRC via interfering tumor immune microenvironment.

**TABLE 3:** Correlation analysis between AZIN1 and various gene markers of immune cells in the TCGA cohort.

Description	Gene markers	KIRC			
		None		Purity	
		Cor	*p*	Cor	*p*
B cell	CD19	0.025	0.572	0.047	0.315
	CD79A	0.012	0.780	0.028	0.546
T cell (general)	CD3D	−0.019	0.659	−0.042	0.370
	CD3E	0.006	0.887	−0.009	0.842
	CD2	0.064	0.143	0.051	0.274
CD4^+^ cell	CD4	0.339	***	0.331	***
CD8^+^ cell	CD8A	0.058	0.184	0.045	0.339
	CD8B	−0.005	0.906	−0.023	0.623
Monocyte	CD86	0.335	***	0.35	***
	CSF1R	0.34	***	0.336	***
M1 Macrophage	NOS2	0.268	***	0.255	***
	IRF5	0.039	0.374	0.004	0.999
	PTGS2	0.307	***	0.347	***
M2 Macrophage	CD163	0.523	***	0.521	***
	VSIG4	0.398	***	0.387	***
	MS4A4A	0.487	***	0.502	***
Neutrophils	CEACAM8	0.05	0.248	0.077	0.0987
	ITGAM	0.334	***	0.336	***
	CCR7	0.145	***	0.157	***
	KIR2DS4	−0.002	0.964	−0.012	0.0797
Dendritic cell	HLA‐DPB1	0.147	***	0.125	**
	HLA‐DQB1	0.043	0.325	0.014	0.761
	HLA‐DRA	0.268	***	0.264	***
	HLA‐DPA1	0.237	***	0.24	***
	CD1C	0.208	***	0.221	***
	NRP1	0.522	***	0.523	***
	ITGAX	0.066	0.130	0.082	0.079

**P < 0.01, ***P < 0.001.

### 3.5 Analysis and prediction of miRNAs of AZIN1

**FIGURE 5:**
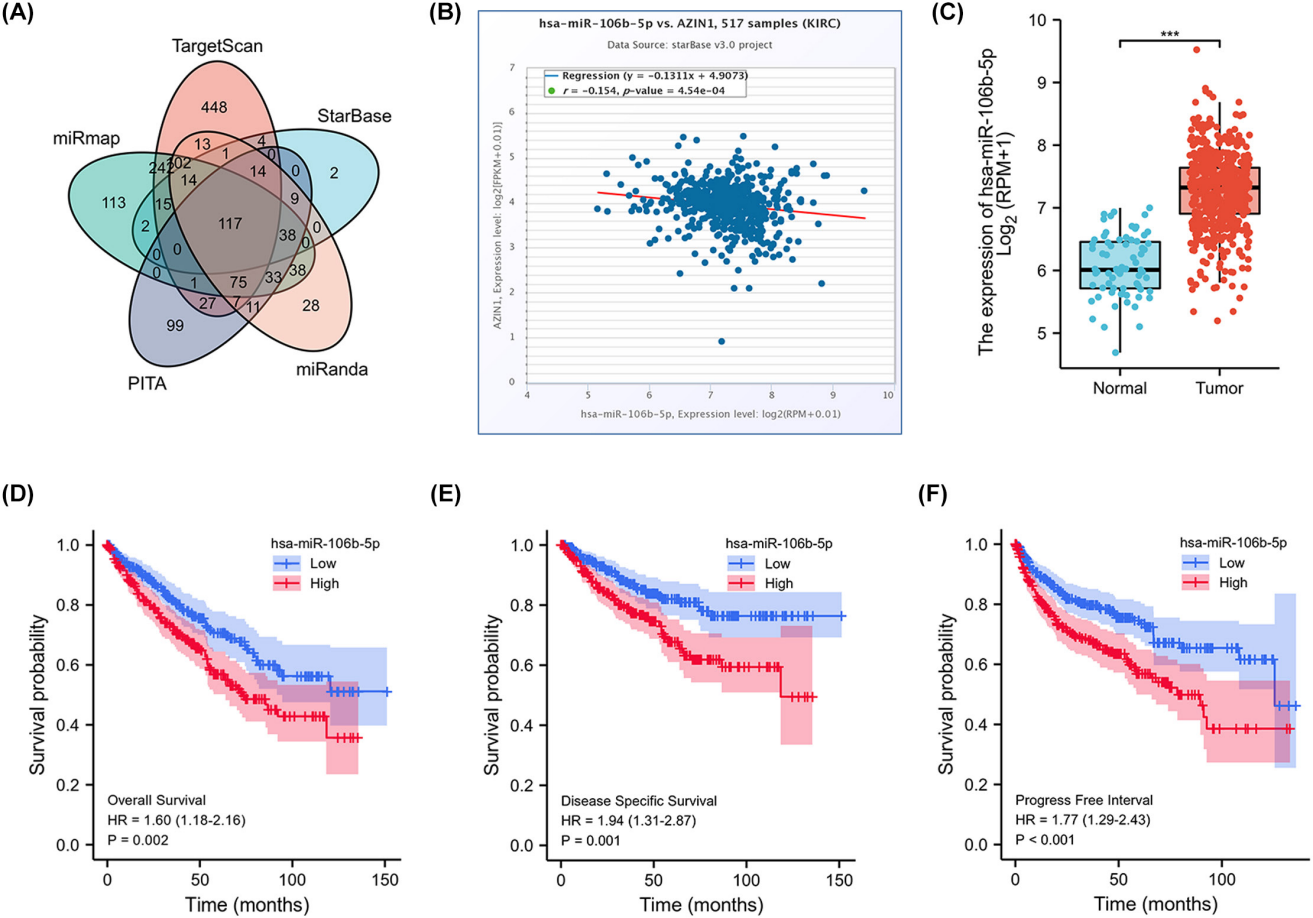
Identification of miR‐106b‐5p as a potential upstream miRNA of Antizyme inhibitor 1 (AZIN1) in kidney renal clear cell carcinoma (KIRC) (A) screening candidate miRNAs of AZIN1 by TargetScan, miRmap, PITA, miRanda, and StarBase. (B) Correlation of miR‐ 106b‐5p and AZIN1 in KIRC by StarBase. (C) miR‐106b‐5p expression in KIRC patients and normal samples in the TCGA database. (D–F) Kaplan–Meier analysis of the association between miR‐106b‐5p expression and overall survival, disease‐free survival, and progression‐free survival. ***p < 0.001.

It is well recognized that non‐coding RNAs (ncRNAs), with no capability in encoding proteins, are highly involved in regulating gene expression. miRNAs are small ncRNAs that generally repress target gene expression.^15^ Dysregulated lncRNAs are also reported to be involved in carcinogenesis in KIRC.^16^ Here, we intended to identify these lncRNA–miRNA regulatory pathways of AZIN1 involved in KIRC patients. First, we analyzed upperstream miRNAs which could potentially bind to AZIN1 via online prediction databases including TargetScan, miRmap, PITA, miRanda and StarBase, and finally found 117 overlapping miRNAs (Figure 5A and Table S2). Among the 117 miRNAs, we found that six miRNAs were negatively correlated with AZIN1 expression from the website (http://starbase.sysu.edu.cn/). The six miRNA levels in KIRC patients were demonstrated in TCGA samples and only miR‐106b‐5p is highly expression in KIRC samples which may lead to the lower expression of AZIN1 in KIRC (Figure S2A and Figure 5B,C). In 517 KIRC samples, miR‐106b‐5 was negatively correlated with AZIN1 expression. The area under the curve was calculated for the diagnostic value of miR‐106b‐5p as indicated in Figure S2B. Moreover, KIRC patients with higher miR‐106b‐5p expression possessed poorer OS, progression‐free survival and disease‐specific survival (Figure 5D–F). According to these results, miR‐106b‐5p might serve as the most potential miRNA that regulates AZIN1 expression in KIRC.

### 3.6 Analysis and prediction of upstream lncRNAs of miR‐106b‐5p

**FIGURE 6:**
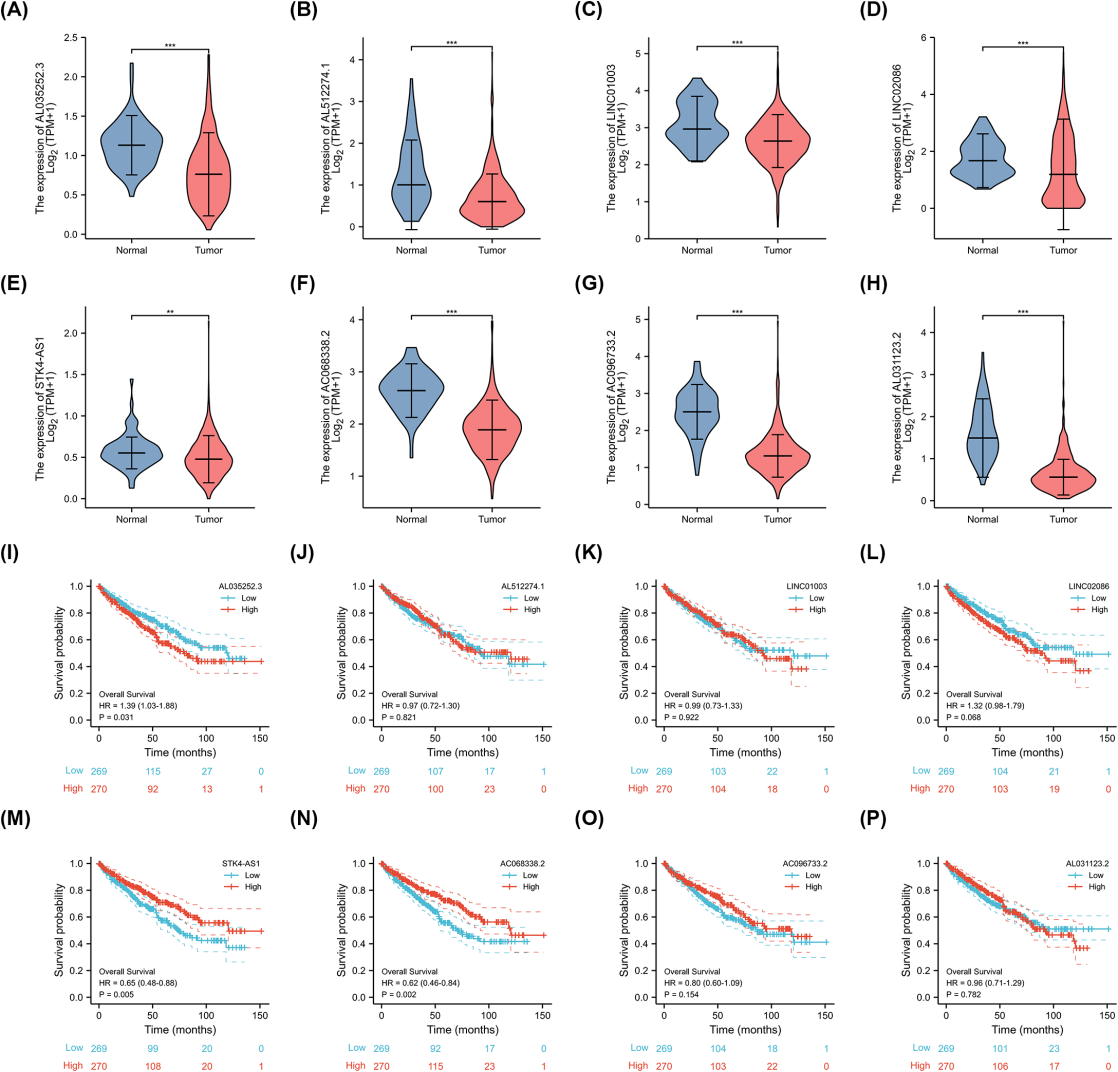
The expression level and OS plots of eight lncRNAs associated with miR‐106b‐5p. (A–H) The expression of eight lncRNAs in kidney renal clear cell carcinoma (KIRC) and control normal samples were determined by the starBase database. (I–P) The association between the expression of eight lncRNAs and overall survival was assessed by the Kaplan–Meier plot.

**FIGURE 7:**
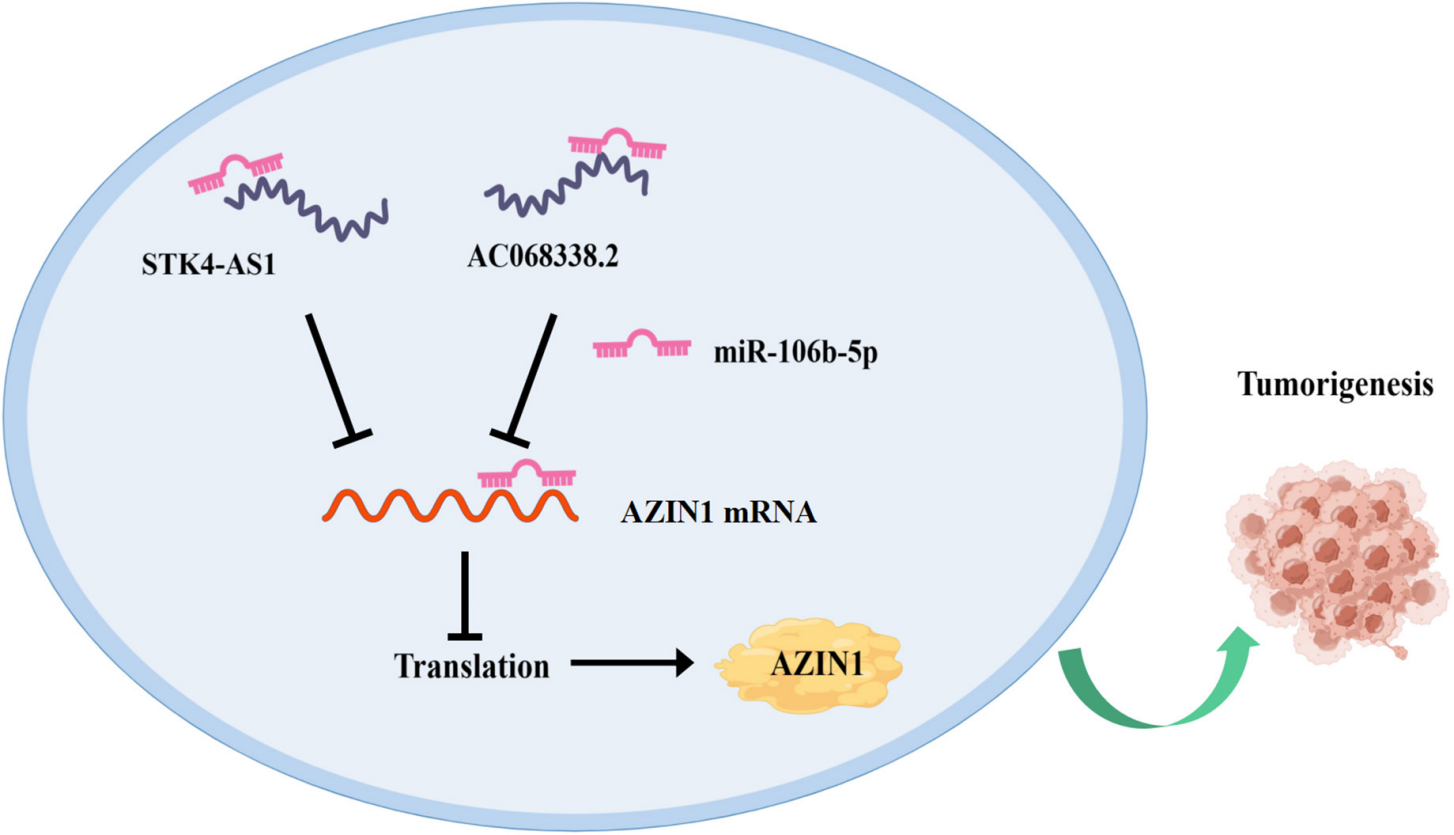
Graphic abstract of STK4‐AS1/AC068338.2‐miR‐106b‐5p‐AZIN1 axis in carcinogenesis of Kidney renal clear cell carcinoma (KIRC).

Next, the upstream lncRNAs of miR‐106b‐5p were predicted using the starBase database. From all the lncRNAs, only eight lncRNAs are downregulated in KIRC samples compared with normal tissues (Figure 6A–H and Table S3). The prognostic analysis of these lncRNAs in KIRC were then assessed. As illustrated in Figure 6I–P, only overexpressed STK4‐AS1 and AC068338.2 indicated a better prognosis. Besides, both of them were independent prognostic factors for OS among KIRC patients (Tables S3 and S4). Given that lncRNA negatively correlated with miRNA in cancer, STK4‐AS1, and AC068338.2 were screened as candidate upstream ncRNAs which negatively regulate miR‐106b‐5p expression in KIRC, suggesting that STK4‐AS1 and AC068338.2 as an independent risk factor and preclinical marker in KIRC and may be associated with KIRC pathogenesis (Tables S4 and S5). Taken above, the graphical representation of STK4‐AS1/AC068338.2‐miR‐106b‐5p‐AZIN1 axis in KIRC is illustrated in Figure 7.

## 4 DISCUSSION

Here, our study first reported the anti‐cancer role of AZIN1 in KIRC. We identified that AZIN1 played a critical role in KIRC among all types of cancers. AZIN1 is downregulated in KIRC and high expression of AZIN1 was associated with favorable outcomes in KIRC patients. Further, AZIN1 performance in immunocytes infiltrated was demonstrated. Finally, we elucidated an upstream regulatory mechanism of AZIN1 in KIRC namely STK4‐AS1/AC068338.2‐miR‐106b‐5p‐AZIN1 axis, as well as the downstream signaling pathways. To our knowledge, this is the first time to illuminate the anti‐oncogenic function of AZIN1 in KIRC.

AZIN1 has been reported to play an essential part in polyamine metabolism. In polyamine biosynthesis, the rate‐limiting process is that Ornithine is converted to putrescene via ODC. Ornithine decarboxylase antizymes (OAZs) usually target ODC for proteasomal degradation. However, AZINs proved to be biochemically similar to ODC, binding to OAZs with higher affinity instead. This rescues ODC activity leading to increased polyamine synthesis. It is worth noting that, between the two isoforms of AZINs, AZIN1 showed increased binding with OAZs than AZIN2.^6^ Taken together, higher expression of AZIN1 is correlated with increased polyamine levels via maintaining the ODC activation.

Polyamines and their metabolites are required for cell proliferation, including the fundamental protein and nucleic acid synthesis as well as cell‐to‐cell communications.^17^ Besides, crosstalks between polyamine metabolism and oncogenic pathways, including RAS‐MAPK, and PI3K–mTOR pathway, have been reported in multiple cancers. Thus, elevated polyamines are usually recognized as biomarkers of cancer. Given the above, AZIN1 acts as a pro‐oncogenic role in most cancers by increasing polyamine levels.^18^

Interestingly, AZIN1 seems to play quite the opposite role in KIRC in this study. Recent data has provided greater insight into the anti‐tumor role of polyamines.

KIRC is an aggressive malignancy characterized by metabolic reprogramming.^7^ The “clear cell” morphology of KIRC tissues is due to abundant lipid and glycogen storage within tumor cells.^19^, ^20^, ^21^, ^22^ Dysregulation of fatty acid synthesis, HMP shunt and glycolysis‐derived metabolites provided an aberrant microenvironment, contributing to tumor progression and poor therapeutic response.^23^, ^24^, ^25^ It's worth noting that AZIN1 A‐to‐I editing, triggered by renal inflammation, not only enhances polyamine biosynthesis but also engages glycolysis and nicotinamide biosynthesis, promoting recovery phenotype.^26^ Yet, limited knowledge of AZIN1's role in glycolysis nicotinamide biosynthesis in KIRC still requires further investigation. It is worth noting that KIRC cells have also been shown to take up excessive levels of polyamines from the tumor microenvironment in hypoxia states.^27^ Dysregulated metabolic and oncogenic signaling pathways also endow tumor cells significantly increasing polyamine synthesis and accumulation even more.^28^ These metabolic changes make KIRC cells particularly vulnerable to polyamine toxicity than other tumor cells.^29^ Furthermore, another research work uncovered that CA9 knockdown led to the accumulation of putrescine which was toxic to KIRC cells, inhibiting KIRC cell growth eventually.^30^ Collectively, these reaffirmed that increased polyamines resulted in the opposite of the Warburg effect and inhibition of tumor cells, which might be the reason for the anti‐tumor role of AZIN1 in KIRC.

As for miRNA, miR‐106b‐5p functions as a pro‐oncogenic role to mediate KIRC cell migration and invasion by targeting PDCD4.^31^ In another study, it has also been shown that downregulation of miR‐106b‐5p made KIRC/CRC‐derived cells more vulnerable to anti‐tumor drugs used in the clinic.^32^ These studies indicated miR‐106b‐5p exerts pro‐oncogenic function in KIRC not only through AZIN1 as a downstream target but also other genes, indicating the pivotal role of miR‐106b‐5p in KIRC, calling for a more comprehensive study.

As for the immune system, polyamines have been widely involved in the activation, proliferation, and differentiation of multiple immune cells.^28^, ^33^ For instance, Tregs and M2‐typed macrophages can facilitate the immunosuppressive microenvironment, contributing to tumor progression.^34^ In addition, KIRC is notably one of the most immune‐infiltrated tumors.^35^, ^36^, ^37^ Emerging evidence indicates that the activation of specific metabolic pathways plays a crucial role in regulating angiogenesis and inflammatory responses.^38^, ^39^ The characteristics of the tumor microenvironment significantly influence disease biology and the efficacy of systemic therapies.^40^, ^41^, ^42^, ^43^, ^44^ Immune dysfunction has been closely linked to cancer progression and prognosis in KIRC.^45^ AZIN1 can modulate immune cell infiltration and regulate immunoflogosis. Features of the tumor microenvironment heavily affect disease biology and may affect responses to systemic therapy. Accumulating data continue to highlight activation of specific metabolic pathway have a pivot role in regulating angiogenesis and inflammatory signatures in KIRC.^38^, ^46^ A recent study proved that tumor‐infiltrating Tregs inhibited anti‐tumor activity in KIRC patients.^47^ In our study, Tregs were highly associated with low expression of AZIN1 in KIRC (Figure 4A). The results also indicated that low levels of AZIN1 predict dismal prognosis in KIRC (Figure 1A,B), which might be associated with Tregs via dampening the immune system in KIRC discussed above.Our studies indicated that the possible mechanism of the anti‐tumor effect of increased AZIN1 and polyamine levels in KIRC might be the result of the production of toxic metabolites as well as their influence on cancer immunity. However, there are still several limitations to this study. First, while our research included comprehensive data analysis, the lack of wet lab experiments conducting functional assays to investigate the effects of AZIN1 modulation on KIRC cell behavior, and validating the proposed regulatory mechanisms involving ncRNAs limits the confirmation and validation of our findings. We just barely scratched the surface of our understanding of the underlying mechanisms of AZIN1 in KIRC. To what extent AZIN1 levels affect polyamines and immune infiltration in KIRC is unclear. Second, lack of basic experiments and animal studies calls for future analysis for validation of expression and prognosis of AZIN1 in KIRC. Further investigations are needed to understand the precise molecular mechanisms which made AZIN1 unique in KIRC, as different from other cancers. Third, instead of ncRNAs, more regulatory mechanisms of AZIN1 in KIRC are worth exploring. Given that multiple biomarkers hold promise for RCC staging and drug efficacy evaluation, the identification of AZIN1 as a potential biomarker presents a new‐emerging target of diagnostic utility and prognostic value, including early detection, risk stratification, and treatment optimization in KIRC patients.

## 5 CONCLUSION

In conclusion, our study provided that AZIN1 is downregulated in KIRC and high levels of AZIN1 were correlated with favorable outcomes among KIRC patients. This study may serve as a starting point for future investigation of AZIN1 in KIRC, providing potential value for guiding immunotherapy and targeted therapy.

## AUTHOR CONTRIBUTIONS

**Xinyi Zeng:** Investigation (equal); writing – original draft (lead); writing – review and editing (equal). **Shen Du:** Formal analysis (lead); investigation (equal); methodology (equal); software (equal). **Lou Geng:** Data curation (equal); investigation (equal); software (equal); supervision (equal); writing – review and editing (equal). **Jiexian Ma:** Funding acquisition (lead); investigation (equal); project administration (equal); supervision (equal); writing – review and editing (equal).

## FUNDING INFORMATION

This work was supported by Shanghai Health Committee (grant number 202140518), the Science and Technology Commission of Shanghai Municipality (grant number 21Y11909000), the Elite Project of Huadong hospital (HD0103).

## CONFLICT OF INTEREST STATEMENT

The authors declare that they have no conflict of interest.

## Supporting information


Figure S1.

Figure S2.

Table S1.

Table S2.

Table S3.

Table S4.

Table S5.

## Data Availability

Data sharing is not applicable to this article as no new data were created or analyzed in this study.
