# Supplementary figures and images for: Nc‐RNA‐mediated low expression of AZIN1 correlated with unfavorable prognosis in kidney renal clear cell carcinoma
Source: Cancer Med. 2024 Aug 14;13(15):e70105. doi: 10.1002/cam4.70105 (PMC11322861; doi:10.1002/cam4.70105)

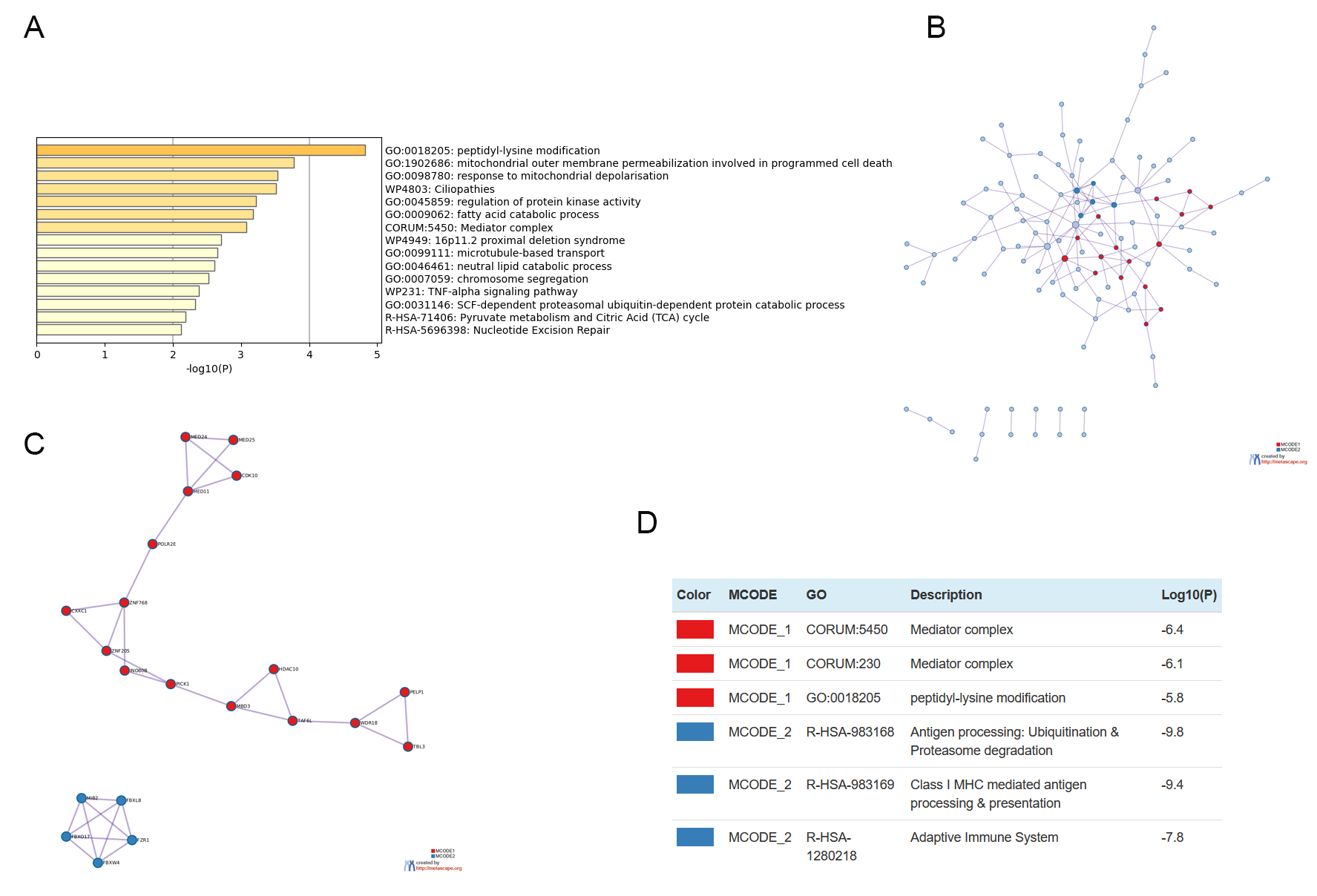

Supplement: Supplementary file 1 — Figure S1. [file CAM4-13-e70105-s002.tif]

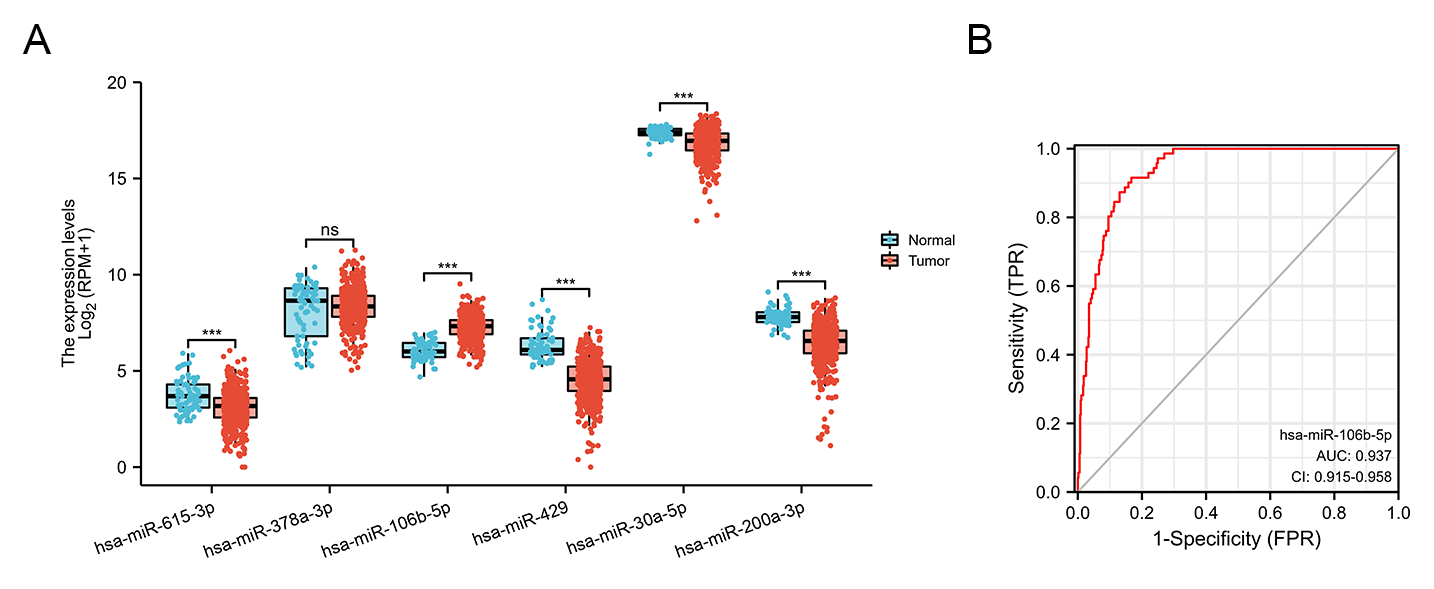

Supplement: Supplementary file 2 — Figure S2. [file CAM4-13-e70105-s003.tif]
