# Supplementary material for: Nc‐RNA‐mediated low expression of AZIN1 correlated with unfavorable prognosis in kidney renal clear cell carcinoma
Source: Cancer Med. 2024 Aug 14;13(15):e70105. doi: 10.1002/cam4.70105 (PMC11322861; doi:10.1002/cam4.70105)
Supplement: Supplementary file 3 — Table S1. [file CAM4-13-e70105-s007.docx]

| **GO** | **Category** | **Description** | **Count** | **%** | **Log10(P)** | **Log10(q)** |
| --- | --- | --- | --- | --- | --- | --- |
| GO:0018205 | GO Biological Processes | peptidyl-lysine modification | 12 | 6.15 | -4.82 | -0.46 |
| GO:1902686 | GO Biological Processes | mitochondrial outer membrane permeabilization involved in programmed cell death | 4 | 2.05 | -3.78 | -0.02 |
| GO:0098780 | GO Biological Processes | response to mitochondrial depolarisation | 3 | 1.54 | -3.53 | -0.01 |
| WP4803 | WikiPathways | Ciliopathies | 7 | 3.59 | -3.51 | -0.01 |
| GO:0045859 | GO Biological Processes | regulation of protein kinase activity | 13 | 6.67 | -3.22 | 0 |
| GO:0009062 | GO Biological Processes | fatty acid catabolic process | 5 | 2.56 | -3.17 | 0 |
| CORUM:5450 | CORUM | Mediator complex | 3 | 1.54 | -3.07 | 0 |
| WP4949 | WikiPathways | 16p11.2 proximal deletion syndrome | 4 | 2.05 | -2.71 | 0 |
| GO:0099111 | GO Biological Processes | microtubule-based transport | 6 | 3.08 | -2.66 | 0 |
| GO:0046461 | GO Biological Processes | neutral lipid catabolic process | 3 | 1.54 | -2.6 | 0 |
| GO:0007059 | GO Biological Processes | chromosome segregation | 8 | 4.1 | -2.52 | 0 |
| WP231 | WikiPathways | TNF-alpha signaling pathway | 4 | 2.05 | -2.38 | 0 |
| GO:0031146 | GO Biological Processes | SCF-dependent proteasomal ubiquitin-dependent protein catabolic process | 3 | 1.54 | -2.32 | 0 |
| R-HSA-71406 | Reactome Gene Sets | Pyruvate metabolism and Citric Acid (TCA) cycle | 3 | 1.54 | -2.18 | 0 |
| R-HSA-5696398 | Reactome Gene Sets | Nucleotide Excision Repair | 4 | 2.05 | -2.12 | 0 |

Table S1. Functional enrichment analysis of negatively correlated genes.
