# Supplementary material for: Nc‐RNA‐mediated low expression of AZIN1 correlated with unfavorable prognosis in kidney renal clear cell carcinoma
Source: Cancer Med. 2024 Aug 14;13(15):e70105. doi: 10.1002/cam4.70105 (PMC11322861; doi:10.1002/cam4.70105)
Supplement: Supplementary file 6 — Table S4. [file CAM4-13-e70105-s006.docx]

| **Characteristics** | **Total(N)** | **Univariate analysis** | |  | **Multivariate analysis** | |
| --- | --- | --- | --- | --- | --- | --- |
|  |  | **Hazard ratio (95% CI)** | **P value** |  | **Hazard ratio (95% CI)** | **P value** |
| Pathologic T stage | 541 |  | **< 0.001** |  |  |  |
| T1&T2 | 350 | Reference |  |  | Reference |  |
| T3&T4 | 191 | 3.210 (2.373 - 4.342) | **< 0.001** |  | 2.207 (1.330 - 3.660) | **0.002** |
| Pathologic N stage | 258 |  | **0.001** |  |  |  |
| N0 | 242 | Reference |  |  | Reference |  |
| N1 | 16 | 3.422 (1.817 - 6.446) | **< 0.001** |  | 0.872 (0.302 - 2.518) | 0.800 |
| Pathologic M stage | 508 |  | **< 0.001** |  |  |  |
| M0 | 429 | Reference |  |  | Reference |  |
| M1 | 79 | 4.401 (3.226 - 6.002) | **< 0.001** |  | 3.756 (2.130 - 6.623) | **< 0.001** |
| Serum calcium | 367 |  | **0.001** |  |  |  |
| Low | 204 | Reference |  |  | Reference |  |
| Normal | 153 | 1.225 (0.865 - 1.735) | 0.254 |  | 0.819 (0.476 - 1.408) | 0.470 |
| Elevated | 10 | 4.846 (2.404 - 9.769) | **< 0.001** |  | 0.760 (0.227 - 2.542) | 0.656 |
| Hemoglobin | 461 |  | **< 0.001** |  |  |  |
| Low | 264 | Reference |  |  | Reference |  |
| Normal | 192 | 0.430 (0.302 - 0.613) | **< 0.001** |  | 0.581 (0.333 - 1.013) | 0.056 |
| Elevated | 5 | 2.663 (0.844 - 8.400) | 0.095 |  | 3.265 (0.418 - 25.522) | 0.259 |
| AC068338.2 | 541 |  | **< 0.001** |  |  |  |
| Low | 270 | Reference |  |  | Reference |  |
| High | 271 | 0.496 (0.364 - 0.677) | **< 0.001** |  | 0.568 (0.340 - 0.948) | **0.030** |

Table S4. Univariate and multivariate Cox regression analyses of AC068338.2 expression and other clinical pathological factors for OS.
