# Supplementary material for: Nc‐RNA‐mediated low expression of AZIN1 correlated with unfavorable prognosis in kidney renal clear cell carcinoma
Source: Cancer Med. 2024 Aug 14;13(15):e70105. doi: 10.1002/cam4.70105 (PMC11322861; doi:10.1002/cam4.70105)
Supplement: Supplementary file 7 — Table S5. [file CAM4-13-e70105-s004.docx]

| **Characteristics** | **Total(N)** | **Univariate analysis** | |  | **Multivariate analysis** | |
| --- | --- | --- | --- | --- | --- | --- |
|  |  | **Hazard ratio (95% CI)** | **P value** |  | **Hazard ratio (95% CI)** | **P value** |
| Pathologic T stage | 541 |  | **< 0.001** |  |  |  |
| T1&T2 | 350 | Reference |  |  | Reference |  |
| T3&T4 | 191 | 3.210 (2.373 - 4.342) | **< 0.001** |  | 2.185 (1.318 - 3.624) | **0.002** |
| Pathologic N stage | 258 |  | **0.001** |  |  |  |
| N0 | 242 | Reference |  |  | Reference |  |
| N1 | 16 | 3.422 (1.817 - 6.446) | **< 0.001** |  | 0.739 (0.225 - 2.421) | 0.617 |
| Pathologic M stage | 508 |  | **< 0.001** |  |  |  |
| M0 | 429 | Reference |  |  | Reference |  |
| M1 | 79 | 4.401 (3.226 - 6.002) | **< 0.001** |  | 4.127 (2.337 - 7.287) | **< 0.001** |
| Serum calcium | 367 |  | **0.001** |  |  |  |
| Low | 204 | Reference |  |  | Reference |  |
| Normal | 153 | 1.225 (0.865 - 1.735) | 0.254 |  | 0.723 (0.423 - 1.237) | 0.237 |
| Elevated | 10 | 4.846 (2.404 - 9.769) | **< 0.001** |  | 0.825 (0.219 - 3.105) | 0.776 |
| Hemoglobin | 461 |  | **< 0.001** |  |  |  |
| Low | 264 | Reference |  |  | Reference |  |
| Normal | 192 | 0.430 (0.302 - 0.613) | **< 0.001** |  | 0.620 (0.355 - 1.084) | 0.094 |
| Elevated | 5 | 2.663 (0.844 - 8.400) | 0.095 |  | 1.961 (0.251 - 15.302) | 0.520 |
| STK4-AS1 | 541 |  | **0.003** |  |  |  |
| Low | 270 | Reference |  |  | Reference |  |
| High | 271 | 0.636 (0.471 - 0.860) | **0.003** |  | 0.535 (0.329 - 0.871) | **0.012** |

Table S5. Univariate and multivariate Cox regression analyses of STK4-AS1 expression and other clinical pathological factors for OS.
